# Supplementary material for: Constitutive deficiency of the neurogenic hippocampal modulator AP2γ promotes anxiety-like behavior and cumulative memory deficits in mice from juvenile to adult periods
Source: eLife. 2021 Dec 3;10:e70685. doi: 10.7554/eLife.70685 (PMC8709574; doi:10.7554/eLife.70685)
Supplement: Figure 1—source data 1. — Western-blot analysis of AP2γ, Sox2, Pax6, and Tbr2 in juvenile (A) and adult (B) dentate gyrus (DG) protein extracts. (C and D) Representative Ponceau staining of membranes corresponding to the juvenile and adult timepoints. Surrounded by orange squares are demonstrated the quantified bands and denoted with a small orange star are identified the bands presented in the main Figure 1. Sample size: Western-blot analysis: nWT juvenile = 4; nAP2γ; KO juvenile = 4; nWT adult = 4; nAP2γ; KO adult = 4; Abbreviations: WT, wild-type; AP2γ KO, AP2γ heterozygous knockout mice. [file elife-70685-fig1-data1.pdf]

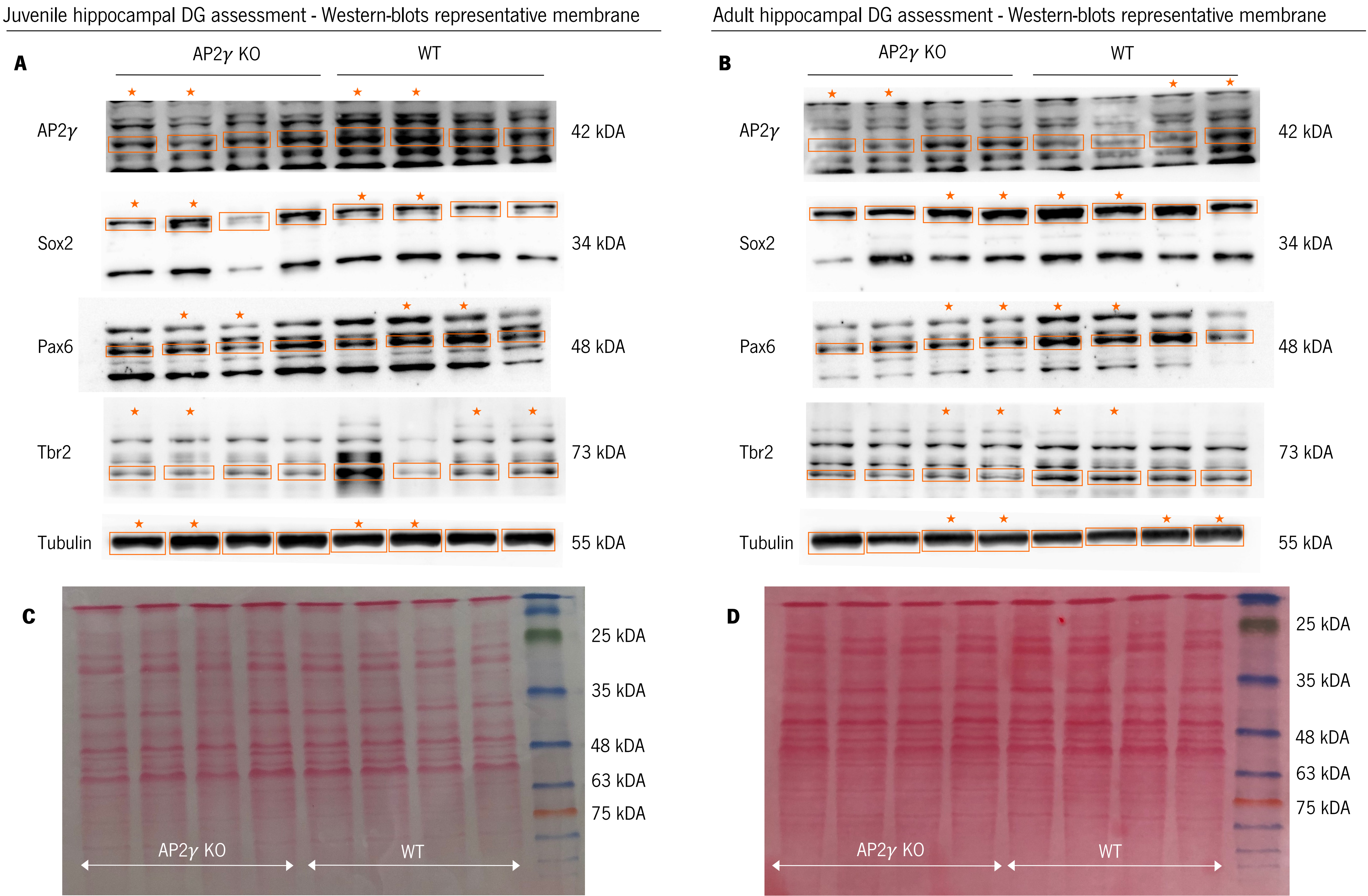
Juvenile hippocampal DG assessment - Western-blots representative membrane
Adult hippocampal DG assessment - Western-blots representative membrane
A
AP2γ KO
WT
AP2γ
42 kDA
Sox2
34 kDA
Pax6
48 kDA
Tbr2
73 kDA
Tubulin
55 kDA
B
AP2γ KO
WT
AP2γ
42 kDA
Sox2
34 kDA
Pax6
48 kDA
Tbr2
73 kDA
Tubulin
55 kDA
C
25 kDA
35 kDA
48 kDA
63 kDA
75 kDA
AP2γ KO
WT
D
25 kDA
35 kDA
48 kDA
63 kDA
75 kDA
AP2γ KO
WT
