# Supplementary material for: Constitutive deficiency of the neurogenic hippocampal modulator AP2γ promotes anxiety-like behavior and cumulative memory deficits in mice from juvenile to adult periods
Source: eLife. 2021 Dec 3;10:e70685. doi: 10.7554/eLife.70685 (PMC8709574; doi:10.7554/eLife.70685)
Supplement: Figure 6—figure supplement 1—source code 1. [file elife-70685-fig2-code2.zip › Figure 6 Figure supplement 1 Source Code 1.docx]

**Figure 6 – Figure supplement 1 – Source Code 1: Local field potentials analysis between the ventral hippocampus (vHip) and medial prefontal cortex (mPFC)**

clc

clear all

%Funcao adequada para os dados da Eduardo Campos (exp.LuísaPinto)

# Initialization of POI Libs

%USAR A FUNCAO XLWRITE EM VEZ DO XLSWRITE QUE SE USA NO WINDOWS

% Add Java POI Libs to matlab javapath

javaaddpath('poi_library/poi-3.8-20120326.jar');

javaaddpath('poi_library/poi-ooxml-3.8-20120326.jar');

javaaddpath('poi_library/poi-ooxml-schemas-3.8-20120326.jar');

javaaddpath('poi_library/xmlbeans-2.3.0.jar');

javaaddpath('poi_library/dom4j-1.6.1.jar');

javaaddpath('poi_library/stax-api-1.0.1.jar');

n_group=2;

n=[10 8]; %no. files in each group

%Output file

output_filename=strcat('LFP_PP_vHip_novo_',datestr(now,'yy-mm-dd_HH-MM-SS'),'.xls');

% Line Filter (50 Hz)

hd=LineFilter;

% Multitaper parameters

tapers=[3 5];

pad=0;

Fs=1000; % FREQUENCIA DE SAMPLING

seg_length=1*Fs;

fpass=[0 100];

err= [2 0.05];

trialave=1;

%Rhythms

Delta=[0.5 4];

%Theta=[4 8];

Theta=[4 12];

Alpha=[8 13];

Beta=[12 20];

Gamma_lo=[20 40];

Gamma_hi=[40 100];

group=[];

coh=[];

psd_ch1=[];

psd_ch2=[];

spect_ch1=[];

spect_ch2=[];

CH1_delta=[];CH1_theta=[];CH1_alpha=[];CH1_beta=[];CH1_gamma_lo=[];CH1_gamma_hi=[];

CH2_delta=[];CH2_theta=[];CH2_alpha=[];CH2_beta=[];CH2_gamma_lo=[];CH2_gamma_hi=[];

CH1_psd_delta=[];CH1_psd_theta=[];CH1_psd_alpha=[];CH1_psd_beta=[];CH1_psd_gamma_lo=[];CH1_psd_gamma_hi=[];

CH2_psd_delta=[];CH2_psd_theta=[];CH2_psd_alpha=[];CH2_psd_beta=[];CH2_psd_gamma_lo=[];CH2_psd_gamma_hi=[];

f_psd = Fs/2*linspace(0,1,Fs/2+1);

Coh_delta=[];Coh_theta=[];Coh_alpha=[];Coh_beta=[];Coh_gamma_lo=[];Coh_gamma_hi=[];

FileName_array={};

for g=1:n_group

data=[];

eval(strcat('[FileName,PathName,FilterIndex]=uigetfile({''.txt''},''Group LFP ',num2str(g),' '',''Multiselect'',''on'');'))

%eval(strcat('[FileName_art,PathName_art]=uigetfile({''.mat''},''Group Artifact Marks ',num2str(g),' '',''Multiselect'',''on'');'))

for i=1:n(g)

file=strcat(PathName,FileName(i));

%file_art=strcat(PathName_art,FileName_art(i));

FileName{i}

%FileName_art{i}

FileName_array=[FileName_array FileName(i)];

[seg]=importdata(cell2mat(file));

%seg(:,1)=seg_i(:,1)*200;

%seg(:,2)=seg_i(:,2)*200;

%[art]=importdata(cell2mat(file_art));

% eval(strcat('file=strcat(PathName,FileName(',num2str(i),'));'))

% eval('[seg]=importdata(file);')

% data(:,2) = mat2gray(data(:,2)) - mean(mat2gray(data(:,2)));

% data(:,3) = mat2gray(data(:,3)) - mean(mat2gray(data(:,3)));

% data=normc(data);

% eval('data=[data;seg];')

%Correct no. columns if necessary

%if (size(seg,2)>2)

% seg(:,1)=[];

%end

% Correct Sampling Rate if necessary

%if (size(seg,1)>25000)

% seg=downsample(seg,4);

%end

seg=filter(hd, seg);

n_seg=size(seg,1)/seg_length;

%exclude segments with artifacts

%markers_ch1=ArtRej_ExtremeValues(seg(:,1),th_PFC(i,1),th_PFC(i,2),200)';

%markers_ch2=ArtRej_ExtremeValues(seg(:,2),th_HIP(i,1),th_HIP(i,2),200)';

%

%art_ch1=zeros(n_seg,1);

%art_ch2=zeros(n_seg,1);

%for s=1:n_seg

% seg_m_ch1=markers_ch1((s-1)*seg_length+1:s*seg_length,1);

% seg_m_ch2=markers_ch2((s-1)*seg_length+1:s*seg_length,1);

% if isempty(find(seg_m_ch1==1))==0

% art_ch1(s)=1;

% end

% if isempty(find(seg_m_ch2==1))==0

% art_ch2(s)=1;

% end

%end

%art_m=art_ch1+art_ch2;

%seg_art=[];

%sg=0;

%art_m=zeros(1,100);

%for s=1:n_seg

% sg=sg+1;

% seg_art(:,sg)=art((sg-1)*seg_length+1:sg*seg_length-1,1);

% if isempty(find(seg_art(:,sg)==1))==0

% art_m(s)=1;

% end

%end

%Separate data in equally sized segments

%seg_ch1=zeros(seg_length,n_seg);

%seg_ch2=zeros(seg_length,n_seg);

seg_ch1=[];

seg_ch2=[];

sg=0;

for s=1:n_seg

%if art_m(s)==0

sg=sg+1;

seg_ch1(:,sg)=seg((sg-1)*seg_length+1:sg*seg_length,1);

seg_ch2(:,sg)=seg((sg-1)*seg_length+1:sg*seg_length,2);

%end

end

%n_seg=sg;

%seg_eliminados=100-sg;

%disp(seg_eliminados)

params=struct('tapers',tapers,'pad',pad,'Fs',Fs,'fpass',fpass,'err',err,'trialave',trialave);

eval(strcat('[C,phi,S_ch1xch2,S_ch1,S_ch2,f,confC,phistd,Cerr]=coherencyc(seg_ch1,seg_ch2,params);'))

%Power Spectral Density

%Pxx_ch1=zeros(Fs,n_seg);

%Pxx_ch2=zeros(Fs,n_seg);

Pxx_ch1=[];

Pxx_ch2=[];

for p=1:n_seg

Pxx_ch1(:,p)= 10*log10( fft(seg_ch1(:,p),Fs).*conj(fft(seg_ch1(:,p),Fs)) );

Pxx_ch2(:,p)= 10*log10( fft(seg_ch2(:,p),Fs).*conj(fft(seg_ch2(:,p),Fs)) );

end

Mean_Pxx_ch1=mean(Pxx_ch1,2);

Mean_Pxx_ch2=mean(Pxx_ch2,2);

delta_psd_i = find(f_psd >= Delta(1) & f_psd < Delta(2));

theta_psd_i = find(f_psd >= Theta(1) & f_psd < Theta(2));

alpha_psd_i = find(f_psd >= Alpha(1) & f_psd < Alpha(2));

beta_psd_i = find(f_psd >= Beta(1) & f_psd < Beta(2));

gamma_lo_psd_i = find(f_psd >= Gamma_lo(1) & f_psd < Gamma_lo(2));

gamma_hi_psd_i = find(f_psd >= Gamma_hi(1) & f_psd < Gamma_hi(2));

CH1_psd_delta=[CH1_psd_delta; mean(Mean_Pxx_ch1(delta_psd_i))];

CH1_psd_theta=[CH1_psd_theta; mean(Mean_Pxx_ch1(theta_psd_i))];

CH1_psd_alpha=[CH1_psd_alpha; mean(Mean_Pxx_ch1(alpha_psd_i))];

CH1_psd_beta=[CH1_psd_beta; mean(Mean_Pxx_ch1(beta_psd_i))];

CH1_psd_gamma_lo=[CH1_psd_gamma_lo; mean(Mean_Pxx_ch1(gamma_lo_psd_i))];

CH1_psd_gamma_hi=[CH1_psd_gamma_hi; mean(Mean_Pxx_ch1(gamma_hi_psd_i))];

CH2_psd_delta=[CH2_psd_delta; mean(Mean_Pxx_ch2(delta_psd_i))];

CH2_psd_theta=[CH2_psd_theta; mean(Mean_Pxx_ch2(theta_psd_i))];

CH2_psd_alpha=[CH2_psd_alpha; mean(Mean_Pxx_ch2(alpha_psd_i))];

CH2_psd_beta=[CH2_psd_beta; mean(Mean_Pxx_ch2(beta_psd_i))];

CH2_psd_gamma_lo=[CH2_psd_gamma_lo; mean(Mean_Pxx_ch2(gamma_lo_psd_i))];

CH2_psd_gamma_hi=[CH2_psd_gamma_hi; mean(Mean_Pxx_ch2(gamma_hi_psd_i))];

% Power Spectrum

eval(strcat('[S_ch1,f,Serr_ch1]=mtspectrumc(detrend(seg_ch1),params);'))

eval(strcat('[S_ch2,f,Serr_ch2]=mtspectrumc(detrend(seg_ch2),params);'))

delta_i = find(f >= Delta(1) & f < Delta(2));

theta_i = find(f >= Theta(1) & f < Theta(2));

alpha_i = find(f >= Alpha(1) & f < Alpha(2));

beta_i = find(f >= Beta(1) & f < Beta(2));

gamma_lo_i = find(f >= Gamma_lo(1) & f < Gamma_lo(2));

gamma_hi_i = find(f >= Gamma_hi(1) & f < Gamma_hi(2));

CH1_delta=[CH1_delta; mean(S_ch1(delta_i))];

CH1_theta=[CH1_theta; mean(S_ch1(theta_i))];

CH1_alpha=[CH1_alpha; mean(S_ch1(alpha_i))];

CH1_beta=[CH1_beta; mean(S_ch1(beta_i))];

CH1_gamma_lo=[CH1_gamma_lo; mean(S_ch1(gamma_lo_i))];

CH1_gamma_hi=[CH1_gamma_hi; mean(S_ch1(gamma_hi_i))];

CH2_delta=[CH2_delta; mean(S_ch2(delta_i))];

CH2_theta=[CH2_theta; mean(S_ch2(theta_i))];

CH2_alpha=[CH2_alpha; mean(S_ch2(alpha_i))];

CH2_beta=[CH2_beta; mean(S_ch2(beta_i))];

CH2_gamma_lo=[CH2_gamma_lo; mean(S_ch2(gamma_lo_i))];

CH2_gamma_hi=[CH2_gamma_hi; mean(S_ch2(gamma_hi_i))];

Coh_delta=[Coh_delta; mean(C(delta_i))];

Coh_theta=[Coh_theta; mean(C(theta_i))];

Coh_alpha=[Coh_alpha; mean(C(alpha_i))];

Coh_beta=[Coh_beta; mean(C(beta_i))];

Coh_gamma_lo=[Coh_gamma_lo; mean(C(gamma_lo_i))];

Coh_gamma_hi=[Coh_gamma_hi; mean(C(gamma_hi_i))];

coh=[coh; C'];

psd_ch1=[psd_ch1;Mean_Pxx_ch1(1:Fs/2+1)'];

psd_ch2=[psd_ch2;Mean_Pxx_ch2(1:Fs/2+1)'];

% spect_ch1=[spect_ch1; (S_ch1/mean(S_ch1(theta_i)))'];

spect_ch1=[spect_ch1; S_ch1'];

% spect_ch2=[spect_ch2; (S_ch2/mean(S_ch2(theta_i)))'];

spect_ch2=[spect_ch2; S_ch2'];

group=[group; g];

end

% Fazer uma discrimina??o das bandas de frequ?ncia

eval(strcat('data',num2str(g),'=data;'))

% eval(strcat('data',num2str(g),'(:,1)=[];'))

%

eval(strcat('data',num2str(g),'=filter(hd, data',num2str(g),');'))

%

% %Separate data in equally sized segments

eval(strcat('n_seg=size(data',num2str(g),',1)/seg_length;'))

eval(strcat('data',num2str(g),'_ch1=zeros(seg_length,n_seg);'))

eval(strcat('data',num2str(g),'_ch2=zeros(seg_length,n_seg);'))

for i=1:n_seg

eval(strcat('data',num2str(g),'_ch1(:,i)=data',num2str(g),'((i-1)*seg_length+1:i*seg_length,1);'))

eval(strcat('data',num2str(g),'_ch2(:,i)=data',num2str(g),'((i-1)*seg_length+1:i*seg_length,2);'))

end

%

params=struct('tapers',tapers,'pad',pad,'Fs',Fs,'fpass',fpass,'err',err,'trialave',trialave);

%

% eval(strcat('[C_',num2str(g),',phi_',num2str(g),',S',num2str(g),'_ch1xch2,S',num2str(g),'_ch1,S',num2str(g),'_ch2,f,confC_',num2str(g),',phistd_',num2str(g),',Cerr_',num2str(g),']=coherencyc(data',num2str(g),'_ch1,data',num2str(g),'_ch2,params);'))

% eval(strcat('[S',num2str(g),'_ch1,f,Serr',num2str(g),'_ch1]=mtspectrumc(detrend(data',num2str(g),'_ch1),params);'))

% eval(strcat('[S',num2str(g),'_ch2,f,Serr',num2str(g),'_ch2]=mtspectrumc(detrend(data',num2str(g),'_ch2),params);'))

end

figure(1)

[Group_Mean,Group_SEM]=grpstats(coh, group, {'mean','std'});

hold on

for g=1:n_group

errorbar(f',Group_Mean(g,:)',Group_SEM(g,:)')

end

xlwrite(output_filename,[{'Freq.'} FileName_array; num2cell(f') num2cell(coh')], 'Coherence')

%title 'Coherency PFC vs Hipp' (canal 1 PFC)

title 'Coherency vHip vs PFC'

xlabel('Frequency (Hz)')

ylabel('Coherency')

hold off

figure(2)

[Group_Mean,Group_SEM]=grpstats(spect_ch1, group, {'mean','std'});

subplot(1,2,1)

hold on

for g=1:n_group

errorbar(f',Group_Mean(g,:)',Group_SEM(g,:)')

end

xlwrite(output_filename,[{'Freq.'} FileName_array; num2cell(f') num2cell(spect_ch1')], 'Power vHip')

%xlwrite(output_filename,[{'Freq.'} FileName_array; num2cell(f') num2cell(spect_ch1')], 'Power PFC')

%title 'Power Frequency Spectrum PFC'

title 'Power Frequency Spectrum vHip'

xlabel('Frequency (Hz)')

ylabel('10*log10(PowerSpectrum)')

hold off

[Group_Mean,Group_SEM]=grpstats(psd_ch1, group, {'mean','std'});

subplot(1,2,2)

hold on

f_psd = Fs/2*linspace(0,1,Fs/2+1);

for g=1:n_group

errorbar(f_psd,Group_Mean(g,:)',Group_SEM(g,:)')

end

xlwrite(output_filename,[{'Freq.'} FileName_array; num2cell((0:1:Fs/2)') num2cell(psd_ch1')], 'PSD vHip')

%xlwrite(output_filename,[{'Freq.'} FileName_array; num2cell((0:1:Fs/2)') num2cell(psd_ch1')], 'PSD PFC')

%title 'Power Spectral Density PFC'

title 'Power Spectral Density vHip'

xlabel('Frequency (Hz)')

ylabel('10*log10(PowerSpectralDensity)')

hold off

figure(3)

[Group_Mean,Group_SEM]=grpstats(spect_ch2, group, {'mean','std'});

subplot(1,2,1)

hold on

for g=1:n_group

errorbar(f',Group_Mean(g,:)',Group_SEM(g,:)')

end

xlwrite(output_filename,[{'Freq.'} FileName_array; num2cell(f') num2cell(spect_ch2')], 'Power PFC')

%xlwrite(output_filename,[{'Freq.'} FileName_array; num2cell(f') num2cell(spect_ch2')], 'Power Hipp')

%title 'Power Frequency Spectrum Hipp'

title 'Power Frequency Spectrum PFC'

xlabel('Frequency (Hz)')

ylabel('10*log10(PowerSpectrum)')

hold off

[Group_Mean,Group_SEM]=grpstats(psd_ch2, group, {'mean','std'});

subplot(1,2,2)

hold on

f_psd = Fs/2*linspace(0,1,Fs/2+1);

for g=1:n_group

errorbar(f_psd,Group_Mean(g,:)',Group_SEM(g,:)')

end

xlwrite(output_filename,[{'Freq.'} FileName_array; num2cell(f_psd') num2cell(psd_ch2')], 'PSD PFC')

%xlwrite(output_filename,[{'Freq.'} FileName_array; num2cell((0:1:125)') num2cell(psd_ch2')], 'PSD Hipp')

%xlwrite(output_filename,[{'Freq.'} FileName_array; num2cell(f_psd') num2cell(psd_ch2')], 'PSD Hipp')

%title 'Power Spectral Density Hipp'

title 'Power Spectral Density PFC'

xlabel('Frequency (Hz)')

ylabel('10*log10(PowerSpectralDensity)')

hold off

figure (4)

hold on

broadband = find(f >= 4 & f <= 40);

%image(f,1:sum(n),coh,'CDataMapping','scaled')

image(f(broadband),1:sum(n),coh(:,broadband),'CDataMapping','scaled')

%title 'Coherency PFC vs Hipp'

title 'Coherency vHip vs PFC'

xlabel('Frequency (Hz)')

ylabel('Frame')

%ylabel('Animals')

hold off

% figure (5)

% subplot(2,2,1) % NAO APARECE???!

% hold on

% image(f(broadband),1:sum(n),spect_ch1,'CDataMapping','scaled')

% %title 'Power PFC'

% title 'Power vHip'

% xlabel('Frequency (Hz)')

% ylabel('Animals')

% hold off

% subplot(2,2,2) % NAO APARECE???!

% hold on

% image(f(broadband),1:sum(n),spect_ch2,'CDataMapping','scaled')

% %title 'Power Hipp'

% title 'Power vHip'

% xlabel('Frequency (Hz)')

% ylabel('Animals')

% hold off

figure (5)

subplot(2,2,1) % NAO APARECE???!

hold on

image(f(broadband),1:sum(n),spect_ch1,'CDataMapping','scaled')

%title 'Power PFC'

title 'Power vHip'

xlabel('Frequency (Hz)')

ylabel('Animals')

hold off

subplot(2,2,2) % NAO APARECE???!

hold on

image(f(broadband),1:sum(n),spect_ch2,'CDataMapping','scaled')

%title 'Power Hipp'

title 'Power vHip'

xlabel('Frequency (Hz)')

ylabel('Animals')

hold off

subplot(1,2,1)

hold on

broadband = find(f_psd >= 4 & f_psd <= 40);

%image(f_psd,1:sum(n),psd_ch1,'CDataMapping','scaled')

image(f_psd(broadband),1:sum(n),psd_ch1(:,broadband),'CDataMapping','scaled')

%title 'PSD PFC'

title 'PSD vHip'

xlabel('Frequency (Hz)')

ylabel('Frame')

%ylabel('Animals')

hold off

subplot(1,2,2)

hold on

%image(f_psd,1:sum(n),psd_ch2,'CDataMapping','scaled')

image(f_psd(broadband),1:sum(n),psd_ch2(:,broadband),'CDataMapping','scaled')

%title 'PSD Hipp'

title 'PSD PFC'

xlabel('Frequency (Hz)')

ylabel('Frame')

%ylabel('Animals')

hold off

figure(6)

for c=1:2

eval(strcat('[Group_Mean_delta,Group_SEM_delta]=grpstats(CH',num2str(c),'_delta, group, {''mean'',''std''});'))

eval(strcat('[Group_Mean_theta,Group_SEM_theta]=grpstats(CH',num2str(c),'_theta, group, {''mean'',''std''});'))

eval(strcat('[Group_Mean_alpha,Group_SEM_alpha]=grpstats(CH',num2str(c),'_alpha, group, {''mean'',''std''});'))

eval(strcat('[Group_Mean_beta,Group_SEM_beta]=grpstats(CH',num2str(c),'_beta, group, {''mean'',''std''});'))

eval(strcat('[Group_Mean_gamma_lo,Group_SEM_gamma_lo]=grpstats(CH',num2str(c),'_gamma_lo, group, {''mean'',''std''});'))

eval(strcat('[Group_Mean_gamma_hi,Group_SEM_gamma_hi]=grpstats(CH',num2str(c),'_gamma_hi, group, {''mean'',''std''});'))

subplot(2,5,(c-1)*5+1) %Delta

hold on

for g=1:n_group

errorbar(g,Group_Mean_delta(g),Group_SEM_delta(g),'x')

end

title 'Delta (0.5-3 Hz)'

ylabel('Power (uV^2)')

xlabel('Group')

hold off

subplot(2,5,(c-1)*5+2) %Theta

hold on

for g=1:n_group

errorbar(g,Group_Mean_theta(g),Group_SEM_theta(g),'x')

end

title 'Theta (4-12 Hz)'

ylabel('Power (uV^2)')

xlabel('Group')

hold off

% subplot(2,6,(c-1)*6+3) %Alpha

% hold on

% for g=1:n_group

% errorbar(g,Group_Mean_alpha(g),Group_SEM_alpha(g),'x')

% end

% title 'Alpha (8-12 Hz)'

% ylabel('Power (uV^2)')

% xlabel('Group')

% hold off

subplot(2,5,(c-1)*5+3) %Beta

hold on

for g=1:n_group

errorbar(g,Group_Mean_beta(g),Group_SEM_beta(g),'x')

end

title 'Beta (13-19 Hz)'

ylabel('Power (uV^2)')

xlabel('Group')

hold off

subplot(2,5,(c-1)*5+4) %Gamma_low

hold on

for g=1:n_group

errorbar(g,Group_Mean_gamma_lo(g),Group_SEM_gamma_lo(g),'x')

end

title 'Low Gamma (20-39 Hz)'

ylabel('Power (uV^2)')

xlabel('Group')

hold off

subplot(2,5,(c-1)*5+5) %Gamma_high

hold on

for g=1:n_group

errorbar(g,Group_Mean_gamma_hi(g),Group_SEM_gamma_hi(g),'x')

end

title 'High Gamma (40-100 Hz)'

ylabel('Power (uV^2)')

xlabel('Group')

hold off

end

xlwrite(output_filename, [{'Animal'} {'Delta'} {'Theta'} {'Alpha'} {'Beta'} {'LoGamma'} {'HiGamma'}; ...

FileName_array' num2cell([CH1_delta CH1_theta CH1_alpha CH1_beta CH1_gamma_lo CH1_gamma_hi])], 'Power Bands vHip')

xlwrite(output_filename,[{'Animal'} {'Delta'} {'Theta'} {'Alpha'} {'Beta'} {'LoGamma'} {'HiGamma'}; ...

FileName_array' num2cell([CH2_delta CH2_theta CH2_alpha CH2_beta CH2_gamma_lo CH2_gamma_hi])], 'Power Bands PFC')

%xlwrite(output_filename, [{'Animal'} {'Delta'} {'Theta'} {'Beta'} {'LoGamma'} {'HiGamma'}; ...

% FileName_array' num2cell([CH1_delta CH1_theta CH1_beta CH1_gamma_lo CH1_gamma_hi])], 'Power Bands vHip')

%xlwrite(output_filename,[{'Animal'} {'Delta'} {'Theta'} {'Beta'} {'LoGamma'} {'HiGamma'}; ...

% FileName_array' num2cell([CH2_delta CH2_theta CH2_beta CH2_gamma_lo CH2_gamma_hi])], 'Power Bands PFC')

figure(7)

for c=1:2

eval(strcat('[Group_Mean_delta,Group_SEM_delta]=grpstats(CH',num2str(c),'_psd_delta, group, {''mean'',''std''});'))

eval(strcat('[Group_Mean_theta,Group_SEM_theta]=grpstats(CH',num2str(c),'_psd_theta, group, {''mean'',''std''});'))

eval(strcat('[Group_Mean_alpha,Group_SEM_alpha]=grpstats(CH',num2str(c),'_psd_alpha, group, {''mean'',''std''});'))

eval(strcat('[Group_Mean_beta,Group_SEM_beta]=grpstats(CH',num2str(c),'_psd_beta, group, {''mean'',''std''});'))

eval(strcat('[Group_Mean_gamma_lo,Group_SEM_gamma_lo]=grpstats(CH',num2str(c),'_psd_gamma_lo, group, {''mean'',''std''});'))

eval(strcat('[Group_Mean_gamma_hi,Group_SEM_gamma_hi]=grpstats(CH',num2str(c),'_psd_gamma_hi, group, {''mean'',''std''});'))

subplot(2,5,(c-1)*5+1) %Delta

hold on

for g=1:n_group

errorbar(g,Group_Mean_delta(g),Group_SEM_delta(g),'x')

end

title 'Delta (0.5-3 Hz)'

ylabel('PSD (uV^2/Hz)')

xlabel('Group')

hold off

subplot(2,5,(c-1)*5+2) %Theta

hold on

for g=1:n_group

errorbar(g,Group_Mean_theta(g),Group_SEM_theta(g),'x')

end

title 'Theta (4-12 Hz)'

ylabel('PSD (uV^2/Hz)')

xlabel('Group')

hold off

% subplot(2,6,(c-1)*6+3) %Alpha

% hold on

% for g=1:n_group

% errorbar(g,Group_Mean_alpha(g),Group_SEM_alpha(g),'x')

% end

% title 'Alpha (8-12 Hz)'

% ylabel('PSD (uV^2/Hz)')

% xlabel('Group')

% hold off

subplot(2,5,(c-1)*5+3) %Beta

hold on

for g=1:n_group

errorbar(g,Group_Mean_beta(g),Group_SEM_beta(g),'x')

end

title 'Beta (13-19 Hz)'

ylabel('PSD (uV^2/Hz)')

xlabel('Group')

hold off

subplot(2,5,(c-1)*5+4) %Gamma_low

hold on

for g=1:n_group

errorbar(g,Group_Mean_gamma_lo(g),Group_SEM_gamma_lo(g),'x')

end

title 'Low Gamma (20-39 Hz)'

ylabel('PSD (uV^2/Hz)')

xlabel('Group')

hold off

subplot(2,5,(c-1)*5+5) %Gamma_high

hold on

for g=1:n_group

errorbar(g,Group_Mean_gamma_hi(g),Group_SEM_gamma_hi(g),'x')

end

title 'High Gamma (40-100 Hz)'

ylabel('PSD (uV^2/Hz)')

xlabel('Group')

hold off

end

xlwrite(output_filename, [{'Animal'} {'Delta'} {'Theta'} {'Alpha'} {'Beta'} {'LoGamma'} {'HiGamma'}; ...

FileName_array' num2cell([CH1_psd_delta CH1_psd_theta CH1_psd_alpha CH1_psd_beta CH1_psd_gamma_lo CH1_psd_gamma_hi])], 'PSD Bands vHip')

xlwrite(output_filename,[{'Animal'} {'Delta'} {'Theta'} {'Alpha'} {'Beta'} {'LoGamma'} {'HiGamma'}; ...

FileName_array' num2cell([CH2_psd_delta CH2_psd_theta CH2_psd_alpha CH2_psd_beta CH2_psd_gamma_lo CH2_psd_gamma_hi])], 'PSD Bands PFC')

%xlwrite(output_filename, [{'Animal'} {'Delta'} {'Theta'} {'Beta'} {'LoGamma'} {'HiGamma'}; ...

% FileName_array' num2cell([CH1_psd_delta CH1_psd_theta CH1_psd_beta CH1_psd_gamma_lo CH1_psd_gamma_hi])], 'PSD Bands PFC')

%xlwrite(output_filename,[{'Animal'} {'Delta'} {'Theta'} {'Beta'} {'LoGamma'} {'HiGamma'}; ...

% FileName_array' num2cell([CH2_psd_delta CH2_psd_theta CH2_psd_beta CH2_psd_gamma_lo CH2_psd_gamma_hi])], 'PSD Bands Hipp')

figure(8)

eval(strcat('[Group_Mean_delta,Group_SEM_delta]=grpstats(Coh_delta, group, {''mean'',''std''});'))

eval(strcat('[Group_Mean_theta,Group_SEM_theta]=grpstats(Coh_theta, group, {''mean'',''std''});'))

eval(strcat('[Group_Mean_alpha,Group_SEM_alpha]=grpstats(Coh_alpha, group, {''mean'',''std''});'))

eval(strcat('[Group_Mean_beta,Group_SEM_beta]=grpstats(Coh_beta, group, {''mean'',''std''});'))

eval(strcat('[Group_Mean_gamma_lo,Group_SEM_gamma_lo]=grpstats(Coh_gamma_lo, group, {''mean'',''std''});'))

eval(strcat('[Group_Mean_gamma_hi,Group_SEM_gamma_hi]=grpstats(Coh_gamma_hi, group, {''mean'',''std''});'))

xlwrite(output_filename,[{'Animal'} {'Delta'} {'Theta'} {'Beta'} {'LoGamma'} {'HiGamma'}; ...

FileName_array' num2cell([Coh_delta Coh_theta Coh_beta Coh_gamma_lo Coh_gamma_hi])], 'Coherence Bands')

subplot(1,5,1) %Delta

hold on

for g=1:n_group

errorbar(g,Group_Mean_delta(g),Group_SEM_delta(g),'x')

end

title 'Delta (0.5-3 Hz)'

ylabel('Coherence (0-1)')

xlabel('Group')

hold off

subplot(1,5,2) %Theta

hold on

for g=1:n_group

errorbar(g,Group_Mean_theta(g),Group_SEM_theta(g),'x')

end

title 'Theta (4-12 Hz)'

ylabel('Coherence (0-1)')

xlabel('Group')

hold off

% subplot(1,6,3) %Alpha

% hold on

% for g=1:n_group

% errorbar(g,Group_Mean_alpha(g),Group_SEM_alpha(g),'x')

% end

% title 'Alpha (8-12 Hz)'

% ylabel('Coherence (0-1)')

% xlabel('Group')

% hold off

subplot(1,5,3) %Beta

hold on

for g=1:n_group

errorbar(g,Group_Mean_beta(g),Group_SEM_beta(g),'x')

end

title 'Beta (13-19 Hz)'

ylabel('Coherence (0-1)')

xlabel('Group')

hold off

subplot(1,5,4) %Gamma Low

hold on

for g=1:n_group

errorbar(g,Group_Mean_gamma_lo(g),Group_SEM_gamma_lo(g),'x')

end

title 'Low Gamma (20-39 Hz)'

ylabel('Coherence (0-1)')

xlabel('Group')

hold off

subplot(1,5,5) %Gamma High

hold on

for g=1:n_group

errorbar(g,Group_Mean_gamma_hi(g),Group_SEM_gamma_hi(g),'x')

end

title 'High Gamma (40-100 Hz)'

ylabel('Coherence (0-1)')

xlabel('Group')

hold off

%ULTIMOS GRAFICOS DO POWER QUE NAO ESTAVAM A SER REPRESENTADOS

% figure (9)

% subplot(1,2,1) % NAO APARECE???!

% hold on

% image(f(broadband),1:sum(n),spect_ch1,'CDataMapping','scaled')

% %title 'Power PFC'

% title 'Power vHip'

% xlabel('Frequency (Hz)')

% ylabel('Animals')

% hold off

% subplot(1,2,2) % NAO APARECE???!

% hold on

% image(f(broadband),1:sum(n),spect_ch2,'CDataMapping','scaled')

% %title 'Power Hipp'

% title 'Power vHip'

% xlabel('Frequency (Hz)')

% ylabel('Animals')

% hold off

% save 'all_animals.mat' group coh spect_ch1 spect_ch2

for i=1:n2 [FileName,PathName,FilterIndex]=uigetfile({'.txt'}, 'Group 2'); file=strcat(PathName,FileName); [data]=importdata(file); % data(:,2) = mat2gray(data(:,2)) - mean(mat2gray(data(:,2))); % data(:,3) = mat2gray(data(:,3)) - mean(mat2gray(data(:,3))); % data=normc(data); data2=[data2;data]; end uigetfile pause time=data1(:,1); data1(:,1)=[]; uiimport pause data2(:,1)=[];

% Line Filter

% hd=LineFilter;

% data1=filter(hd, data1);

% data2=filter(hd, data2);

% data1(:,1) = mat2gray(data1(:,1)) - mean(mat2gray(data1(:,1)));

% data1(:,2) = mat2gray(data1(:,2)) - mean(mat2gray(data1(:,2)));

% data2(:,1) = mat2gray(data2(:,1)) - mean(mat2gray(data2(:,1)));

% data2(:,2) = mat2gray(data2(:,2)) - mean(mat2gray(data2(:,2)));

% tapers=[3 5];

% pad=0;

% Fs=250;

% fpass=[0 100];

% err= [2 0.05];

% trialave=1;

%Separate data in equally sized segments

% seg_length=2*Fs;

% n_seg=size(data1,1)/seg_length;

% data1_ch1=zeros(seg_length,n_seg);

% data1_ch2=zeros(seg_length,n_seg);

% for i=1:n_seg

% data1_ch1(:,i)=data1((i-1)*seg_length+1:i*seg_length,1);

% data1_ch2(:,i)=data1((i-1)*seg_length+1:i*seg_length,2);

% end

%

% n_seg=size(data2,1)/seg_length;

% data2_ch1=zeros(seg_length,n_seg);

% data2_ch2=zeros(seg_length,n_seg);

% for i=1:n_seg

% data2_ch1(:,i)=data2((i-1)*seg_length+1:i*seg_length,1);

% data2_ch2(:,i)=data2((i-1)*seg_length+1:i*seg_length,2);

% end

% params=struct('tapers',tapers,'pad',pad,'Fs',Fs,'fpass',fpass,'err',err,'trialave',trialave);

%

% [C_init,phi_init,Sinit_ch1xch2,Sinit_ch1,Sinit_ch2,f,confC_init,phistd_init,Cerr_init]=coherencyc(data1_ch1,data1_ch2,params);

% [C_end,phi_end,Send_ch1xch2,Send_ch1,Send_ch2,f,confC_end,phistd_end,Cerr_end]=coherencyc(data2_ch1,data2_ch2,params);

% figure(2)

% % eval(strcat('fill([f f(end:-1:1)],[Cerr_1(1,:) Cerr_1(2,end:-1:1)],[0.5 0.5 0.1])'))

% hold on

% for g=1:n_group

% eval(strcat('fill([f f(end:-1:1)],[Cerr_',num2str(g),'(1,:) Cerr_',num2str(g),'(2,end:-1:1)],[0.5 0.5 0.5])'))

% end

% for g=1:n_group

% eval(strcat('plot(f,C_',num2str(g),',''-b'',''LineWidth'',1)'))

% end

% % plot(f,C_end,'-k','LineWidth',1)

% title 'Coherency PFC vs Hipp'

% xlabel('Frequency (Hz)')

% ylabel('Coherency')

% hold off

%

% % [S1_ch1,f,Serr1_ch1]=mtspectrumc(detrend(data1_ch1),params);

% % [S1_ch2,f,Serr1_ch2]=mtspectrumc(detrend(data1_ch2),params);

% % [S2_ch1,f,Serr2_ch1]=mtspectrumc(detrend(data2_ch1),params);

% % [S2_ch2,f,Serr2_ch2]=mtspectrumc(detrend(data2_ch2),params);

%

% figure(3)

% % fill([f f(end:-1:1)],[10*log10(Serr1_ch1(1,:)) 10*log10(Serr1_ch1(2,end:-1:1))],[1 0.5 0.5])

% hold on

% for g=1:n_group

% eval(strcat('fill([f f(end:-1:1)],[10*log10(Serr',num2str(g),'_ch1(1,:)) 10*log10(Serr',num2str(g),'_ch1(2,end:-1:1))],[0.5 0.5 0.5])'))

% eval(strcat('plot(f,10*log10(S',num2str(g),'_ch1),''-r'',''LineWidth'',1)'))

% end

% % plot(f,10*log10(S2_ch1),'-k','LineWidth',1)

% title 'PFC init vs end'

% xlabel('Frequency (Hz)')

% ylabel('10*log10(PowerSpectrum)')

% hold off

%

% figure(4)

% % fill([f f(end:-1:1)],[10*log10(Serr1_ch2(1,:)) 10*log10(Serr1_ch2(2,end:-1:1))],[1 0.5 0.2])

% hold on

% for g=1:n_group

% eval(strcat('fill([f f(end:-1:1)],[10*log10(Serr',num2str(g),'_ch2(1,:)) 10*log10(Serr',num2str(g),'_ch2(2,end:-1:1))],[0.5 0.5 0.5])'))

% eval(strcat('plot(f,10*log10(S',num2str(g),'_ch2),''-w'',''LineWidth'',1)'))

% end

% % plot(f,10*log10(S2_ch2),'-k','LineWidth',1)

% title 'Hipp init vs end'

% xlabel('Frequency (Hz)')

% ylabel('10*log10(PowerSpectrum)')

% hold off
