# Supplementary material for: Constitutive deficiency of the neurogenic hippocampal modulator AP2γ promotes anxiety-like behavior and cumulative memory deficits in mice from juvenile to adult periods
Source: eLife. 2021 Dec 3;10:e70685. doi: 10.7554/eLife.70685 (PMC8709574; doi:10.7554/eLife.70685)
Supplement: Supplementary file 1. [file elife-70685-supp1.docx]

**Supplementary file 1:**

**Table 1:** Statistical summary of results

| **Experiment** | **Figure(s)** | **Statistical details** |
| --- | --- | --- |
| **Western blot protein quantifications** | **Figure 1C** | **AP2γ quantification:**  Student’s t-test, *t*_6=_ 3.26, *p*< 0.01  **Sox2 quantification:**  Student’s t-test, *t_6_*= 0.33, *p*= 0.75  **Pax6 quantification:**  Student’s t-test, *t_6_*= 2.58, *p*< 0.05  **Tbr2 quantification:**  Student’s t-test, *t_6_*= 2.93, *p*< 0.05  n_WT juvenile_ = 4; n_AP2γ KO juvenile_ = 4 |
|  | **Figure 1D** | **AP2γ quantification:**  Student’s t-test, *t*_6=_ 3.95, *p*< 0.01  **Sox2 quantification:**  Student’s t-test, *t_6_*= 1.41, *p*= 0.21  **Pax6 quantification:**  Student’s t-test, *t_6_*= 4.67, *p*< 0.01  **Tbr2 quantification:**  Student’s t-test, *t_6_*= 2.56, *p*< 0.05  n_WT adult_ = 4; n_AP2γ KO adult_ = 4 |
| **Cell proliferation** | **Figure 1F** | **BrdU^+^ cells:**  Student’s t-test, *t_10_*= 2.47, *p*< 0.05  n_WT juvenile_ = 6; n _AP2γ KO juvenile_ = 6; |
|  | **Figure 1G** | **BrdU^+^DCX^+^cells:**  Student’s t-test, *t_10_*= 2.58, p< 0.05  n_WT juvenile_ = 6; n_AP2γ KO juvenile_ = 6; |
|  | **Figure 1H** | **BrdU^+^ cells:**  Student’s t-test, *t_8_*= 2.67, p< 0.05  n_WT adult_ = 5; n_AP2γ KO adult_ = 5 |
|  | **Figure 1I** | **BrdU^+^DCX^+^cells:**  Student’s t-test, ^+^: *t_8_*= 3.53, p< 0.01  n_WT adult_ = 5; n_AP2γ KO adult_ = 5 |
|  | **Juvenile vs Adult comparison** | **BrdU^+^ cells:**  Two-way ANOVA, *F*_(1,18)_= 147.8, p<0.001  Bonferroni’s multiple comparisons test:  Juvenile _WT vs_ Adult _WT_: p<0.001  Juvenile _AP2γ KO vs_ Adult _AP2γ KO_: p<0.001  n_WT juvenile_ = 6; n_WT juvenile_ = 5  n_AP2γKO juvenile_ = 6; n_AP2γKO adult_ = 5 |
|  | **Juvenile vs Adult Comparison** | **BrdU^+^DCX^+^cells:**  Two-way ANOVA, *F*_(1,18)_= 186.5, p<0.001  Bonferroni’s multiple comparisons test:  Juvenile _WT vs_ Adult _WT_: p<0.001  Juvenile _AP2γ KO vs_ Adult _AP2γ KO_: p<0.001  n_WT juvenile_ = 6; n_WT juvenile_ = 5  n_AP2γ KO juvenile_ = 6; n_AP2γ KO adult_ = 5 |

| **DCX^+^ cells density and morphology** | **Figure 2C** | **Short-DCX^+^ cells density:**  Student’s t-test, *t*_6_= 3.53, *p<*0.05  n_WT adult_ = 4; n_AP2γ KO adult_ = 4 |
| --- | --- | --- |
|  | **Figure 2E** | **Dendritic length of short-DCX^+^ cells:**  Student’s t-test, *t*_6_= 5.54, *p<*0.01  n_WT adult_ = 4; n_AP2γ KO adult_ = 4 |
|  | **Figure 2F** | **Neuronal arborization:**  Genotype’s comparison (WT vs AP2γ KO):  Repeated measures ANOVA, F_(1,78)_= 70.09, *p<*0.001  n_WT adult_= 4  n_AP2γ KO adult_ = 4 |
|  | **Figure 2G** | **Long-DCX^+^ cells density:**  Student’s t-test, *t*_6_= 5.87, *p<*0.01  n_WT adult_ = 4; n_AP2γ KO adult_ = 4 |
|  | **Figure 2I** | **Dendritic length of long-DCX^+^ cells:**  Student’s t-test, *t*_6_= 0.05, *p*= 0.96  n_WT adult_ = 4; n_AP2γ KO adult_ = 4 |
|  | **Figure 2J** | **Neuronal arborization:**  Genotype’s comparison (WT vs AP2γ KO):  Repeated measures ANOVA, F_(1,78)_= 2.47, *p=* 0.12  n_WT adult_= 4  n_AP2γ KO adult_ = 4 |
| **3D neuronal reconstruction** | **Figure 2 – Supplement 1A** | **Dendritic length:**  Student’s t-test, *t*_6_= 0.29, *p*= 0.78  n_WT juvenile_ = 4; n_AP2γ KO juvenile_ = 4 |
|  | **Figure 2 – Supplement 1B** | **Dendritic length:**  Student’s t-test, *t*_7_= 0.06, *p*= 0.96  n_WT adult_ = 4; n_AP2γ KO adult_ = 5 |
|  | **Figure 2 – Supplement 1C** | **Neuronal arborization:**  Genotype’s comparison (WT vs AP2γ KO):  Juvenile phase:  Repeated measures ANOVA, F_(1,72)_= 1.20, *p*= 0.28  Adulthood:  Repeated measures ANOVA, F_(1,84)_= 1.12, *p*= 0.29  n_WT juvenile_ = 4; n_AP2γ KO juvenile_= 4  n_WT adult_ _juvenile_ = 5; n_AP2γ KO adult_ = 5 |

| **OF**  (juvenile) | **Figure 3B; Figure 3 – Supplement 2A** | **Distance in center:**  Student’s t-test, *t*_22_ = 2.64, *p*<0.05  **Average velocity:**  Student’s t-test, *t_22_*= 0.40, *p*= 0.69  n_WT_ = 13; n_AP2γ KO_ = 11 |
| --- | --- | --- |
| **NSF**  (juvenile) | **Figure 3C and D; Figure 3 Supplement 2B** | **Latency to touch:**  Student’s t-test, *t*_26_ = 2.06, *p*<0.05  **Latency to eat:**  Student’s t-test, *t*_26_ = 2.00, *p =* 0.05  **Food consumption:**  Repeated measures ANOVA, F_(1,31)_= 0.07, *p=* 0.80  n_WT_ = 16; n_AP2γ KO_ = 17 |
| **TST**  (juvenile) | **Figure 3E** | **Immobility time:**  Student’s t-test, *t_27_*= 0.23, *p*= 0.82  n_WT_ = 16; n_AP2γ KO_ = 13 |
| **SST**  (juvenile) | **Figure 3F** | **Grooming time:**  Student’s t-test, *t_23_*= 0.05, *p*= 0.96  n_WT_ = 15; n_AP2γ KO_ = 10 |
| **MWM**  (juvenile) | **Figure 3G** | **Spatial Reference memory task:**  Repeated measures ANOVA, F_(1,17)_= 0.27, *p*= 0.61 |
| **ORT**  (juvenile) | **Figure 3I and J** | **Object location exploration:**  Student’s t-test, *t_17_*= 0.79, *p*=0.27  **Object recognition exploration:**  Student’s t-test, *t_17_*= 3.33, *p*<0.05  n_WT_ = 11; n_AP2γ KO_ = 8 |
| **OIC**  (juvenile) | **Figure 3L** | **Patter separation:**  Two-way ANOVA, *F*_(1,62)_= 15.26, p<0.001  Bonferroni’s multiple comparisons test:  WT Familiar Context vs WT Out of Context: p<0.001  AP2γ KO Familiar Context vs AP2γ KO Out of Context:  p = 0.94  WT Out of Context vs AP2γ KO Out of Context: p<0.01  n_WT_ = 16; n_AP2γ KO_ = 17 |
| **CFC**  (juvenile) | **Figure 3N – Q** | **Before Conditioning:**  Student’s t-test, *t_31_*= 0.56, *p*= 0.58  **Context probe A:**  Student’s t-test, *t_31_*= 3.60, *p*< 0.01  **Context probe B:**  Student’s t-test, *t_31_*= 0.70, *p*= 0.49  **Cue Probe:**  Student’s t-test, *t_3_*_1_= 1.0.76, *p*= 0.45  n_WT_ = 16; n_AP2γ KO_ = 17 |

| **OF**  (adult) | **Figure 4B and Figure 4 – supplement 1A** | **Distance in center:**  Student’s t-test, *t_24_*= 2.10, *p*= 0.05  **Average velocity:**  Student’s t-test, *t*_24_=0.49, *p*= 0.63  n_WT_ = 12; n_AP2γ KO_ = 14 |
| --- | --- | --- |
| **EPM**  (adult) | **Figure 4C** | **Open arms time:**  Student’s t-test, *t*_18_= 3.10, *p*< 0.01  n_WT_ = 12; n_AP2γ KO_ = 14 |
| **NSF**  (adult) | **Figure 4D and E; Figure 4 Supplement 1B** | **Latency to touch:**  Student’s t-test, *t_17_* = 3.66, *p*<0.01  **Latency to eat:**  Student’s t-test, *t_17_* = 2.76, *p*<0.01  **Food consumption:**  Repeated measures ANOVA, F_(1,19)_= 0.28, *p=* 0.60  n_WT_ = 10; n_AP2γ KO_= 9 |
| **FST**  (adult) | **Figure 4F** | **Immobility time:**  Student’s t-test, *t*_24_= 1.26, *p*= 0.22  n_WT_ = 12; n_AP2γ KO_ = 14 |
| **TST**  (adult) | **Figure 4G** | **Immobility time:**  Student’s t-test, *t*_10_= 0.64, *p*= 0.54  n_WT_ = 6; n_AP2γ KO_ = 6 |
| **ORT**  (adult) | **Figure 4H and I** | **Object location exploration:**  Student’s t-test, *t_19_*= 1.80, *p*= 0.08  **Object recognition exploration:**  Student’s t-test; *t_19_*= 0.26, *p*= 0.79  n_WT_ = 12; n_AP2γ KO_ = 9 |
| **PS**  (adult) | **Figure 4J** | **Patter separation:**  Two-way ANOVA, *F*_(1,40)_= 21.94, p<0.001  Bonferroni’s multiple comparisons test:  WT Familiar Context vs WT Out of Context: *p*<0.001  AP2γ KO Familiar Context vs AP2γ KO Out of Context:  *p* = 0.18  WT Out of Context vs AP2γ KO Out of Context: *p*<0.05  n_WT_ = 12; n_AP2γ KO_ = 10 |
| **CFC**  (adult) | **Figure 4K – M** | **Before Conditioning:**  Student’s t-test, *t_11_*= 0.38, *p*= 0.71  **Context probe A:**  Student’s t-test, *t*_11_= 2.84, *p*< 0.05  **Context probe B:**  Student’s t-test, *t*_11_= 0.34, *p*= 0.75  **Cue Probe:**  Student’s t-test, *t*_11_= 1.26, *p*= 0.24  n_WT_ = 7; n_AP2γ KO_ = 6 |
| **MWM**  (adult) | **Figure 5B and 5C; Figure 5 – Supplement 1** | **Spatial Reference memory task:**  Repeated measures ANOVA, F_(1,72)_= 1.35, *p*= 0.25  **Behavior flexibility:**  Student’s t-test, *t*_18_= 6.79, *p* <0.001  **Working memory task:**  Repeated measures ANOVA, F_(1,72)_= 0.85, *p*= 0.36  n_WT_ = 10; n_AP2γ KO_ = 10 |

| **Spectral coherence**  **dHip-mPFC** | **Figure 6B** | Two-way ANOVA, *F*_(1,9)_= 7.32, p< 0.05  Bonferroni’s multiple comparisons test:  **Delta:** WT vs AP2γ KO *p* < 0.05  **Theta:** WT vs AP2γ KO *p* < 0.05  **Beta:** WT vs AP2γ KO *p* < 0.05  **Low gamma:** WT vs AP2γ KO *p* = 0.11  **High gamma:** WT vs AP2γ KO *p* = 0.80  n_WT_ = 6; n_AP2γ KO_ = 5 |
| --- | --- | --- |
| **PSD values**  **dHIP** | **Figure 6C** | Two-way ANOVA, *F*_(1,9)_= 3.20, *p* = 0.09  Bonferroni’s multiple comparisons test:  **Delta:** WT vs AP2γ KO *p* = 0.94  **Theta:** WT vs AP2γ KO *p* = 0.07  **Beta:** WT vs AP2γ KO *p* = 0.08  **Low gamma:** WT vs AP2γ KO *p* = 0. 52  **High gamma:** WT vs AP2γ KO *p* = 0.99  n_WT_ = 6; n_AP2γ KO_ = 5 |
| **PSD values**  **mPFC** | **Figure 6D** | Two-way ANOVA, *F*_(1,9)_= 8.64, p< 0.05  Bonferroni’s multiple comparisons test:  **Delta:** WT vs AP2γ KO *p* < 0.05  **Theta:** WT vs AP2γ KO *p* < 0.05  **Beta:** WT vs AP2γ KO *p* < 0.05  **Low gamma:** WT vs AP2γ KO *p* = 0.08  **High gamma:** WT vs AP2γ KO *p* = 0.60  n_WT_ = 6; n_AP2γ KO_ = 5 |
| **Spectral coherence**  **vHIP-mPFC** | **Figure 6 – Figure 6 supplement 1B** | Two-way ANOVA, *F*_(1,8)_= 0.02, *p* = 0.88  Bonferroni’s multiple comparisons test:  **Delta:** WT vs AP2γ KO *p* = 0.59  **Theta:** WT vs AP2γ KO *p* = 0.90  **Beta:** WT vs AP2γ KO *p* = 0.99  **Low gamma:** WT vs AP2γ KO *p* = 0.98  **High gamma:** WT vs AP2γ KO *p* = 0.90  n_WT_ = 5; n_AP2γ KO_ = 5 |
| **PSD values**  **vHIP** | **Figure 6 – Figure 6 supplement 1C** | Two-way ANOVA, *F*_(1,8)_= 2.96, *p* = 0.12  Bonferroni’s multiple comparisons test:  **Delta:** WT vs AP2γ KO *p* = 0.09  **Theta:** WT vs AP2γ KO *p* = 0.77  **Beta:** WT vs AP2γ KO p> 0.99  **Low gamma:** WT vs AP2γ KO p > 0.99  **High gamma:** WT vs AP2γ KO p = 0.86  n_WT_ = 5; n_AP2γ KO_ = 5 |
