# Supplementary material for: Constitutive deficiency of the neurogenic hippocampal modulator AP2γ promotes anxiety-like behavior and cumulative memory deficits in mice from juvenile to adult periods
Source: eLife. 2021 Dec 3;10:e70685. doi: 10.7554/eLife.70685 (PMC8709574; doi:10.7554/eLife.70685)
Supplement: Transparent reporting form [file elife-70685-transrepform1.pdf]

Westbrook Centre
Milton Road
Cambridge CB4 1YG, UK

P 01223 855340
W elifesciences.org
T @elife

eLife's transparent reporting form

We encourage authors to provide detailed information *within their submission* to facilitate the interpretation and replication of experiments. Authors can upload supporting documentation to indicate the use of appropriate reporting guidelines for health-related research (see EQUATOR Network), life science research (see the BioSharing Information Resource), or the ARRIVE guidelines for reporting work involving animal research. Where applicable, authors should refer to any relevant reporting standards documents in this form.

If you have any questions, please consult our Journal Policies and/or contact us: editorial@elifesciences.org.

Sample-size estimation

- You should state whether an appropriate sample size was computed when the study was being designed
- You should state the statistical method of sample size computation and any required assumptions
- If no explicit power analysis was used, you should describe how you decided what sample (replicate) size (number) to use

Please outline where this information can be found within the submission (e.g., sections or figure legends), or explain why this information doesn't apply to your submission:

eLife Sciences Publications, Ltd is a limited liability non-profit non-stock corporation incorporated in the State of Delaware, USA, with company number 5030732, and is registered in the UK with company number FC030576 and branch number BR015634 at the address Westbrook Centre, Milton Road, Cambridge CB4 1YG.

Protein quantification. We did not calculate statistical power prior to performing the western blots; we used a number of animals similar to previous studies (n= 4) (Mateus Pinheiro et al., 2016) and 2 to 3 technical replicates to ensure reliability of the data.
Statistical method used: Data normality was assessed through the Shapiro-Wilk statistical test considering the respective histograms and measures of skewness and kurtosis. When the normality was reached, we used Student's t-test, and the Mann-Whitney when the normality of data was not verified.

Immunostaining and 3D neuronal reconstruction analysis. We did not calculate statistical power before these analyses; we used a number of animals similar to previous studies (n=4-6) (Alves et al., 2017; Mateus Pinheiro et al., 2016; Patricio et al., 2015), 5 slides of each animal for cell proliferation counting's, and 10 neurons for dendritic length and morphology analysis for each animal.
Statistical method used: When the distribution was normal, we applied the Student's t-test, and the Mann-Whitney when the normality of data was not verified.

Behavioral testing. The sample size for behavioral testing was chosen considering an effect size of 0.8, α error probability of 0.05, and the power of 0.7. When the statistical method applied was the student's t-test the total sample size obtained from the calculation was n=32, and when it was needed the repeated measures ANOVA the sample size obtained was n=24. Due to the high number of cohorts and timepoints analyzed in this study, in some cases, these high sample sizes were not feasible. However, previous studies in the field using similar sample sizes were taken into consideration (Alves et al., 2017; Mateus Pinheiro et al., 2016; Patricio et al., 2015). All sample sizes are discriminated in all figure's legends, and in the statistical summary tables (Table 1 and Supplementary Table 1).

Electrophysiological recordings. Statistical power was not defined prior to electrophysiological assessments. However, we had in consideration previous studies that used similar sample sizes (Sardinha et al., 2017; Mateus Pinheiro et al., 2016; Oliveira et al., 2013).
Statistical method used: Repeated Measures ANOVA was applied when normality was observed, and the Mann-Whitney when the normality of data was not verified. All sample sizes are discriminated at the respective figure's legends and in the statistical summary table (Supplementary Table 1).

Calculations for sample number estimation were done using G*Power software (University Kiel, Germany). For all experiments, information about the sample size is provided in the results section, figures and legends, and summarized in Table 1 and Supplementary Table 1.

Replicates

- You should report how often each experiment was performed
- You should include a definition of biological versus technical replication
- The data obtained should be provided and sufficient information should be provided to indicate the number of independent biological and/or technical replicates
- If you encountered any outliers, you should describe how these were handled
- Criteria for exclusion/inclusion of data should be clearly stated

eLife Sciences Publications, Ltd is a limited liability non-profit non-stock corporation incorporated in the State of Delaware, USA, with company number 5030732, and is registered in the UK with company number FC030576 and branch number BR015634 at the address Westbrook Centre, Milton Road, Cambridge CB4 1YG.

- High-throughput sequence data should be uploaded before submission, with a private link for reviewers provided (these are available from both GEO and ArrayExpress)

Please outline where this information can be found within the submission (e.g., sections or figure legends), or explain why this information doesn't apply to your submission:

Biological replication – replicates were performed for different biological samples, when performing protein quantification, immunostainings and 3D neuronal reconstructions, or different animals, in the case of behavioral tests and electrophysiological recordings.

Technical replication – repetition of the procedure with the same sample was only performed for protein quantification, immunostainings and 3D neuronal reconstructions.

All behavioral tests were performed at different time points of analyses, using for that independent cohorts of animals. All tests were performed in at least two independent sets of animals. The electrophysiological recordings were acquired in one set of animals.

For exclusion of outliers, we used the ROUT statistical method.

All this information can be found at the Material and Methods section, in all Figures and Figures legends, in Table 1 and in the Supplementary File 1.

Statistical reporting

- Statistical analysis methods should be described and justified
- Raw data should be presented in figures whenever informative to do so (typically when N per group is less than 10)
- For each experiment, you should identify the statistical tests used, exact values of N, definitions of center, methods of multiple test correction, and dispersion and precision measures (e.g., mean, median, SD, SEM, confidence intervals; and, for the major substantive results, a measure of effect size (e.g., Pearson's r, Cohen's d)
- Report exact p-values wherever possible alongside the summary statistics and 95% confidence intervals. These should be reported for all key questions and not only when the p-value is less than 0.05.

Please outline where this information can be found within the submission (e.g., sections or figure legends), or explain why this information doesn't apply to your submission:

eLife Sciences Publications, Ltd is a limited liability non-profit non-stock corporation incorporated in the State of Delaware, USA, with company number 5030732, and is registered in the UK with company number FC030576 and branch number BR015634 at the address Westbrook Centre, Milton Road, Cambridge CB4 1YG.

(For large datasets, or papers with a very large number of statistical tests, you may upload a single table file with tests, Ns, etc., with reference to sections in the manuscript.)

All statistical methods are described in the Materials and Methods section. Statistical analysis was based on the number of samples, groups and number of variables in study. We followed the data normality through the Shapiro-Wilk test, and when data followed the Gaussian curve we applied parametric tests such as the Student's T-test and ANOVA tests, while in non-normal data we used the corresponding non-parametric statistical tests. All this information is presented and summarized in Supplementary Table 1.

For all graphs, we provide information about the statistical test used, the number of biological samples or animals, methods of multiple test correction, and the format of data presentation (dispersion measures: either mean ± SEM or median ± Interquartile range).

Data and statistical details:

Protein quantification. We used the student's t-test and the results are presented in Figure 1, and the detailed statistical information including effect size and p value is summarized in Supplementary Table 1.

Assessment of cell proliferation. We used both student's -test and also Two-way ANOVA depending on statistical comparisons. These results are presented in Figure 1 and in the Supplementary Figure 1. In all figure legends and in the supplementary table 1 we included the statistical test applied, effect size and p value.

3D neuronal reconstruction analysis. We used the student's t-test, Two-way ANOVA and repeated measures ANOVA depending on statistical comparisons. The results are presented in Figure 1 and Supplementary figure 1. Detailed statistical information including effect size and p value is summarized in Supplementary Table 1.

Behavioral analysis. Results of behavioral tests are presented in Figures and Supplementary Figures 2, 3 and 4. Distinct statistical tests were used depending on the variables in study. For the vast majority, the students' t-test and the Repeated measures ANOVA were applied. Statistical information including effect size and p value is summarized in Table 1 and Supplementary Table 1.

Electrophysiological assessment. Data from electrophysiological recordings is presented in Figure and Supplementary Figure 5. For the analysis of the spectral coherence and of the power spectral densities of the recorded brain areas we used the student's t-test. Detailed statistical information including effect size and p value is summarized in Supplementary Table 1.

For all analysis, we included in the corresponding figure legend and in the presented tables all required statistical details (statistical tests used, test correction, effect size, p values, and detailed statistical values according to the performed statistical method).

eLife Sciences Publications, Ltd is a limited liability non-profit non-stock corporation incorporated in the State of Delaware, USA, with company number 5030732, and is registered in the UK with company number FC030576 and branch number BR015634 at the address Westbrook Centre, Milton Road, Cambridge CB4 1YG.

Group allocation

- Indicate how samples were allocated into experimental groups (in the case of clinical studies, please specify allocation to treatment method); if randomization was used, please also state if restricted randomization was applied
- Indicate if masking was used during group allocation, data collection and/or data analysis

Please outline where this information can be found within the submission (e.g., sections or figure legends), or explain why this information doesn't apply to your submission:

All littermate mice used in this study were allocated according to their genotype, and different cohorts of animals were used at the different timepoints of analysis.

During analysis, experimenters were blind to the genotype of biological samples or animals included in the study.

Additional data files ("source data")

- We encourage you to upload relevant additional data files, such as numerical data that are represented as a graph in a figure, or as a summary table
- Where provided, these should be in the most useful format, and they can be uploaded as "Source data" files linked to a main figure or table
- Include model definition files including the full list of parameters used
- Include code used for data analysis (e.g., R, MatLab)
- Avoid stating that data files are "available upon request"

Please indicate the figures or tables for which source data files have been provided:

For the analysis of electrophysiological recordings, we used available and previously published toolboxes (Sardinha et al., 2017; Mateus Pinheiro et al., 2016; Oliveira et al., 2013). However, attached to the submission files we added the MATLAB scripts used.
